# Impairment of microbial and meiofaunal ecosystem functions linked to algal forest loss

**DOI:** 10.1038/s41598-020-76817-5

**Published:** 2020-11-17

**Authors:** Silvia Bianchelli, Roberto Danovaro

**Affiliations:** 1 Dipartimento di Scienze della Vita e dell’Ambiente, Università Politecnica delle Marche, Via Brecce Bianche, 60131 Ancona, Italy; 2 Stazione Zoologica Anton Dohrn di Napoli, Villa Comunale, 80121 Naples, Italy

**Keywords:** Biodiversity, Ecology, Restoration ecology, Ecosystem ecology, Marine biology

## Abstract

Habitat loss is jeopardizing marine biodiversity. In the Mediterranean Sea, the algal forests of *Cystoseira* spp. form one of the most complex, productive and vulnerable shallow-water habitats*.* These forests are rapidly regressing with negative impact on the associated biodiversity, and potential consequences in terms of ecosystem functioning. Here, by comparing healthy *Cystoseira* forests and barren grounds (i.e., habitats where the macroalgal forests disappeared), we assessed the effects of habitat loss on meiofaunal and nematode biodiversity, and on some ecosystem functions (here measured in terms of prokaryotic and meiofaunal biomass). Overall, our results suggest that the loss of *Cystoseira* forests and the consequent barren formation is associated with the loss of meiofaunal higher taxa and a decrease of nematode biodiversity, leading to the collapse of the microbial and meiofaunal variables of ecosystem functions. We conclude that, given the very limited resilience of these ecosystems, active restoration of these vulnerable habitats is needed, in order to recover their biodiversity, ecosystem functions and associated services.

## Introduction

Human alteration of ecosystems and particularly habitat degradation, fragmentation and loss are causing widespread biodiversity loss and the decline of important ecosystem functions, leading to reduced provision of goods and services^1–5^. The loss of habitat-forming species is of primary concern, as ecosystem engineers modify the environment, providing secondary substrates, which support the co-existence of a large biodiversity and biomass^6–9^, and sustain high levels of productivity and other ecosystem functions^10–12^.

The current global decline in the abundances of canopy-forming macroalgae^13^ is causing the loss of some of the most diverse and productive ecosystems in shallow coastal hard-bottom ecosystems^14^. Global and local stressors have been identified as the main drivers of macroalgal forest declines, from global climate change (shifts in bioclimatic zones and ocean acidification), to the presence of invasive species, from trampling to eutrophication^15,16^. Several studies have investigated the mechanisms causing shifts from macroalgal forests to barren grounds or algal turfs^9^. The decline of macroalgal forests has also been widely documented in the Mediterranean Sea: 9 species of Fucales were reported 25 years ago, and only 5 species of the 14 reported in early 1900s are now present in the NW Mediterranean^17,18^. Some macroalgal species commonly reported in the past are now rare or even locally extinct^17^. Some studies have documented wide fluctuations in the abundances of the canopy-forming seaweeds over more than a century^19^, while others have reported a high temporal stability of the canopy of *Cystoseira* and *Sargassum* forests^20^, including of the species *Cystoseira amentacea*, regarded as a highly vulnerable and threatened species^19^.

Although a partial recovery of *Cystoseira* forests has been reported from the western coast of the Adriatic Sea^21^, and the Fucales are still diversified and stable^18–23^ there have been dramatic losses of 50 to > 80% of *Cystoseira* forests in the Mediterranean Sea (Danovaro, unpublished data). *Cystoseira* species are listed as “of community interest” according to the Habitat Directive (92/43/EEC)^24^ and are indicators of environmental quality in Mediterranean coastal waters according to the Water Framework Directive (2000/60/EC)^25–28^. Several species of this genus are protected by the “Convention on the Conservation of European Wildlife and Natural Habitats” (i.e., Bern Convention), recognized as a priority by the Barcelona Convention and considered vulnerable by international organizations (i.e. IUCN, RAC/SPA, MedPan).

In terms of their contributions to ecosystem functions, seaweed-dominated nearshore habitats have been ranked among the most relevant carbon sinks (ca 173 TgC year^−1^ sequestration^29^), leading to so called “blue carbon strategies”, aiming at exploring the potential of vegetated-marine habitats for mitigating climate change^30–32^. Due to their relevance in the provision of ecosystem services^15^, the magnitude of macroalgal forests decline is leading the scientific community to investigate the best strategies for their protection, as well as exploring the most efficient tools for their restoration^33–35^.

The effects of degradation and/or fragmentation of macrophyte habitats have thus far been only marginally assessed^36–38^, and information on the impacts of biodiversity loss on ecosystem functioning and efficiency are mostly confined to effects on primary production, sea urchins and fishes^34,39,40^. The relationships between biodiversity and ecosystem functioning can vary amongst ecosystems and regions^41–46^, and researchers have also investigated the relationships between macrofaunal biodiversity and ecosystem functions associated with macroalgae^34,39,40^. Conversely, limited information is available on the effects of habitat loss on prokaryotes and meiofauna, which play key roles in ecosystem functioning, biogeochemical cycles, and energy transfer in food webs^47,48^.

The functioning of marine ecosystems dominated by primary producers (e.g., seagrasses, mangroves) is largely controlled by the availability of inorganic nutrients and thus on the rates of organic matter cycling. These depend on heterotrophic prokaryotic production and on the amounts of available organic matter^48^. In these systems, organic detritus is converted into prokaryotic biomass and then, through the microbial food web, enters higher trophic levels, passing through different trophic levels, including the small-size components inhabiting the sediments, such as meiofauna^47–49^.

In the present study we investigated the relationships between biodiversity and ecosystem functioning and efficiency comparing macroalgal forests and barren grounds (areas where macroalgal forests are absent, and possibly have been lost) in six areas of the Mediterranean Sea. In particular, we tested the hypothesis that some ecosystem functions (measured as organic matter degradation rates, prokaryotic and meiofaunal biomass^47–52^) change between macroalgal forests and barrens. We also evaluated associated changes in biodiversity, including diversity at higher taxonomic levels as well as nematode diversity and life-history strategies.

## Results

### Ecosystem functioning in *Cystoseira* spp. forests and barren grounds

**Figure 1:**
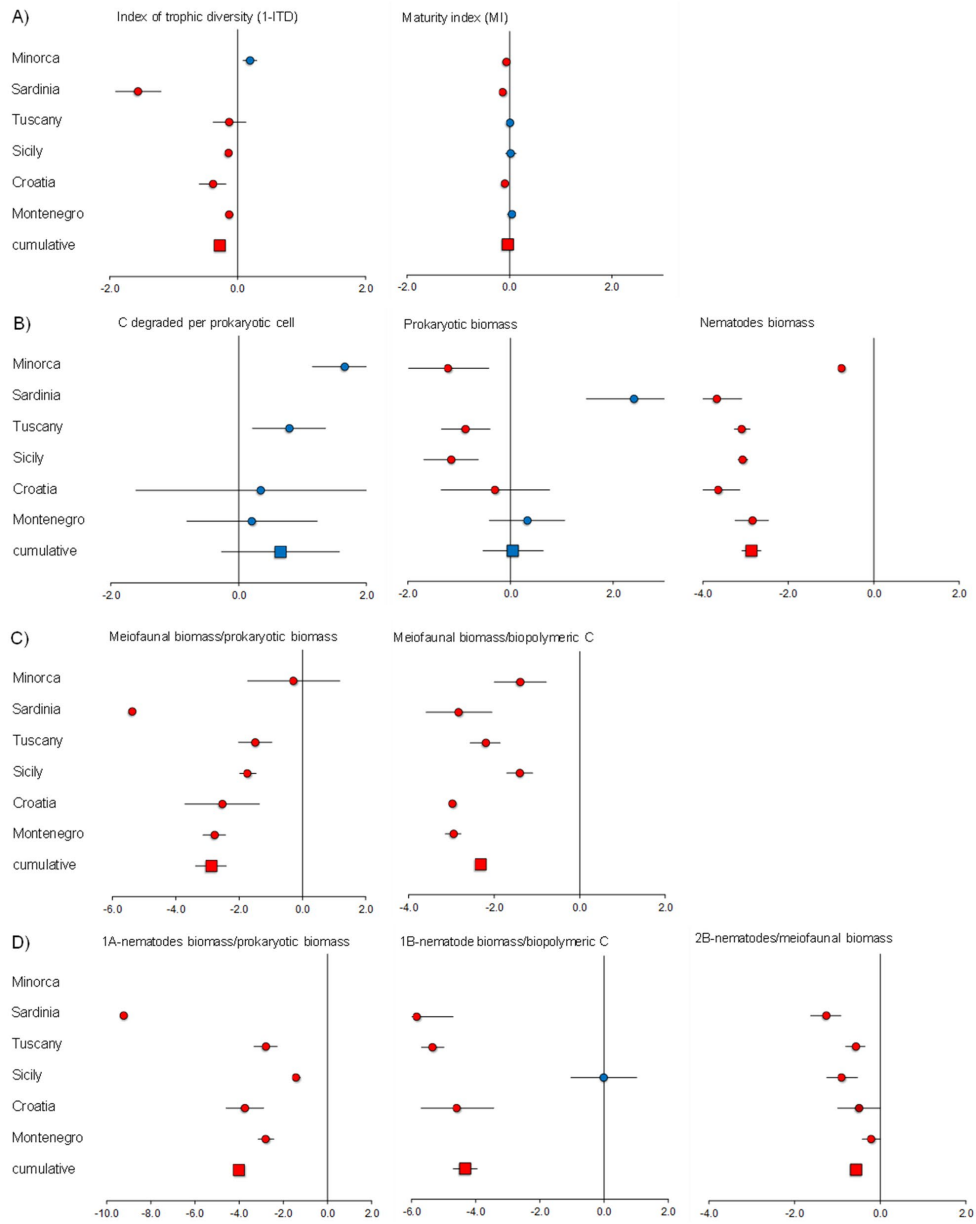
Forest plots showing the negative effects (red dots) of barren grounds on nematode trophic diversity and life strategies (**A**), ecosystem functioning (**B**) and efficiency at meiofaunal higher taxonomic (**C**) and nematode species level (**D**) in all investigated areas (round symbols) and cumulatively for all areas (square symbols). Bars represent the standard error.

The variables used as proxies of ecosystem functioning (i.e., degraded C per prokaryotic cell, prokaryotic and nematode biomass) in macroalgal forests and barren grounds in all the investigated areas are reported in Table 1. These results were compared with data on meiofaunal variables (i.e., richness of meiofaunal higher taxa, expected species number ES(51), index of trophic diversity 1-ITD and maturity index MI and sedimentary organic matter, measured as biopolymeric C concentration, BPC) previously reported from the same areas^36^ (Fig. 1A).

**Table 1:** C degraded per prokaryotic cell, prokaryotic and nematode biomass in the investigated sites and areas (*na *data not available).

	C degradated per prokaryotic cell		Prokaryotic biomass		Nematode biomass	
	µgC cell^−1^ h^−1^		µgC g^−1^		µgC 10 cm^−2^	
	avg	sd	avg	sd	avg	sd
**Minorca**						
Barren						
Site 1	3.1E−06	7.1E−07	4.0	4.9E−01	0.7	0.2
Site 2	1.5E−06	1.0E−07	12.1	4.6E−01	0.7	0.4
Forest						
Site 1	4.1E−07	1.2E−07	23.3	3.6E−01	1.4	0.5
Site 2	na	na	na		1.6	0.2
**Sardinia**						
Barren						
Site 1	na	na	20.0	1.2E+00	0.2	0.1
Site 2	na	na	10.5	3.2E−01	0.1	0.1
Forest						
Site 1	na	na	2.7	3.0E−01	9.8	1.9
Site 2	na	na	0.6	2.9E−02	3.7	1.8
**Tuscany**						
Barren						
Site 1	8.6E−07	3.3E−07	2.7	2.5E−02	0.3	0.1
Site 2	7.4E−07	1.8E−07	4.0	1.0E−01	0.2	0.1
Forest						
Site 1	2.2E−07	1.1E−07	11.2	1.9E+00	4.7	1.7
Site 2	5.9E−07	3.0E−07	5.5	9.7E−01	5.5	1.0
**Sicily**						
Barren						
Site 1	na	na	2.4	8.2E−02	0.1	0.0
Site 2	na	na	2.2	6.5E−02	0.0	0.0
Forest						
Site 1	na	na	11.5	4.1E−01	1.1	0.1
Site 2	na	na	4.6	1.8E−01	1.1	0.2
**Croatia**						
Barren						
Site 1	2.3E−06	1.1E−06	3.8	2.8E−01	0.2	0.1
Site 2	8.7E−08	2.9E−08	22.7	1.2E+00	0.5	0.1
Forest						
Site 1	2.2E−07	9.5E−08	14.9	1.2E+00	15.3	2.6
Site 2	4.7E−07	2.6E−08	10.4	6.1E−01	9.8	2.3
**Montenegro**						
Barren						
Site 1	1.9E−06	4.2E−07	5.5	3.8E−01	0.2	0.1
Site 2	1.7E−06	5.4E−07	6.1	1.8E−01	0.4	0.1
Forest						
Site 1	6.0E−07	6.7E−08	7.9	6.4E−01	4.9	1.4
Site 2	3.5E−06	7.6E−07	2.2	9.8E−02	4.6	0.9

The results of PERMANOVA analyses, testing for the factors State (i.e., forest vs barren) and Site (i.e., between random sites within each state), on the investigated variables measuring the ecosystem functioning are reported in Supplementary Table S1-A.

The factor State had no significant effect, whereas the factor Site had a significant effect on C degraded per prokaryotic cell in three areas (for two areas the data were not available). The factor Site had a significant effect also on prokaryotic biomass at all areas.

The factor State had a significant effect on nematodes biomass, with lower values in barren grounds than in forests, in all investigated areas. In two areas, also the factor Site had a significant effect on nematode biomass. The forest plots illustrated the results (Fig. 1B).

Data dealing with variables used as proxies of the ecosystem efficiency in macroalgal forests and barren grounds in all the investigated areas (i.e., meiofaunal biomass to prokaryotic biomass and BPC ratios, 1A-nematodes(bacterivorous) to prokaryotic biomass, 1B (i.e., detritivorous)—nematodes biomass to BPC and 2B (predators)—nematodes to meiofaunal biomass ratios) are reported in Table 2. The results of PERMANOVA analyses on the ecosystem efficiency variables are reported in Supplementary Table S1-B.

**Table 2:** Meiofaunal/prokaryotic biomass ratio, meiofaunal biomass/biopolymeric C ratio, 1A-nematodes/prokaryotic biomass ratio, 1B-nematodes biomass/biopolymeric C and 2B-nematodes/ meiofaunal biomass ratio in all investigated ares (*na *data not available).

	Meiofaunal biomass/prokaryotic biomass	Meiofaunal biomass/biopolymeric C	1A-nematodes biomass/prokaryotic biomass	1B-nematodes biomass/biopolymeric C	2B-nematodes biomass/meiofaunal biomass
**Minorca**					
Barren					
Site 1	0.32	1.03E−04	na	na	na
Site 2	0.04	1.86E−04	na	na	na
Forest					
Site 1	0.04	4.37E−05	1.04E−05	0.00E+00	0.01
Site 2	0.04	4.81E−05	0.00E+00	1.58E−08	0.00
**Sardinia**					
Barren					
Site 1	0.39	8.99E−05	1.50E−03	1.48E−08	0.02
Site 2	0.23	1.09E−04	5.97E−04	1.14E−08	0.01
Forest					
Site 1	0.13	3.77E−05	1.76E−04	0.00E+00	0.01
Site 2	0.11	9.03E−05	0.00E+00	3.07E−07	0.01
**Tuscany**					
Barren					
Site 1	0.29	3.81E−05	1.09E−03	1.27E−07	0.01
Site 2	0.04	6.13E−05	2.63E−04	3.77E−08	0.03
Forest					
Site 1	0.10	2.51E−05	5.77E−04	0.00E+00	0.03
Site 2	0.13	4.98E−05	1.66E−03	0.00E+00	0.02
**Sicily**					
Barren					
Site 1	0.15	1.82E−04	na	na	na
Site 2	Na	1.13E−03	na	na	na
Forest					
Site 1	9.22	1.74E−03	6.74E−02	8.75E−07	0.02
Site 2	8.07	4.63E−04	1.13E−01	3.06E−06	0.02
**Croatia**					
Barren					
Site 1	0.49	4.70E−04	1.13E−02	1.25E−06	0.03
Site 2	1.94	1.42E−03	1.52E−02	3.59E−06	0.02
Forest					
Site 1	0.35	1.68E−04	5.90E−04	9.29E−08	0.02
Site 2	0.84	3.46E−04	6.34E−04	1.22E−07	0.02
**Montenegro**					
Barren					
Site 1	0.97	1.16E−03	2.72E−02	1.45E−05	0.02
Site 2	1.28	1.10E−03	1.86E−02	3.09E−06	0.03
Forest					
Site 1	0.96	6.60E−04	1.71E−02	1.86E−06	0.03
Site 2	2.93	7.75E−04	1.78E−02	9.11E−06	0.02

The State had a significant effect on meiofaunal/prokaryotes biomass and meiofaunal biomass/BPC ratios, in almost all investigated areas, with higher values reported in forests than in barren grounds. The State had a significant effect on 1A-nematodes/prokaryotic biomass ratio in all areas and on 1B-nematodes biomass/BPC ratio in almost all areas. In both cases, higher values were reported in *Cystoseria* spp. forests than in barren grounds. The State had a significant effect on 2B-nematodes/meiofaunal biomass ratio at three areas, with higher values in *Cystoseria* spp. forests. The forest plots showed that barren grounds had significant negative effects on all the variables used as proxies of ecosystem efficiency, with only one exception where a null effect was observed (in Sicily, for the 1B-nematode biomass/biopolymeric C ratio; Fig. 1C,D).

### Effects of biodiversity on ecosystem functioning

**Figure 2:**
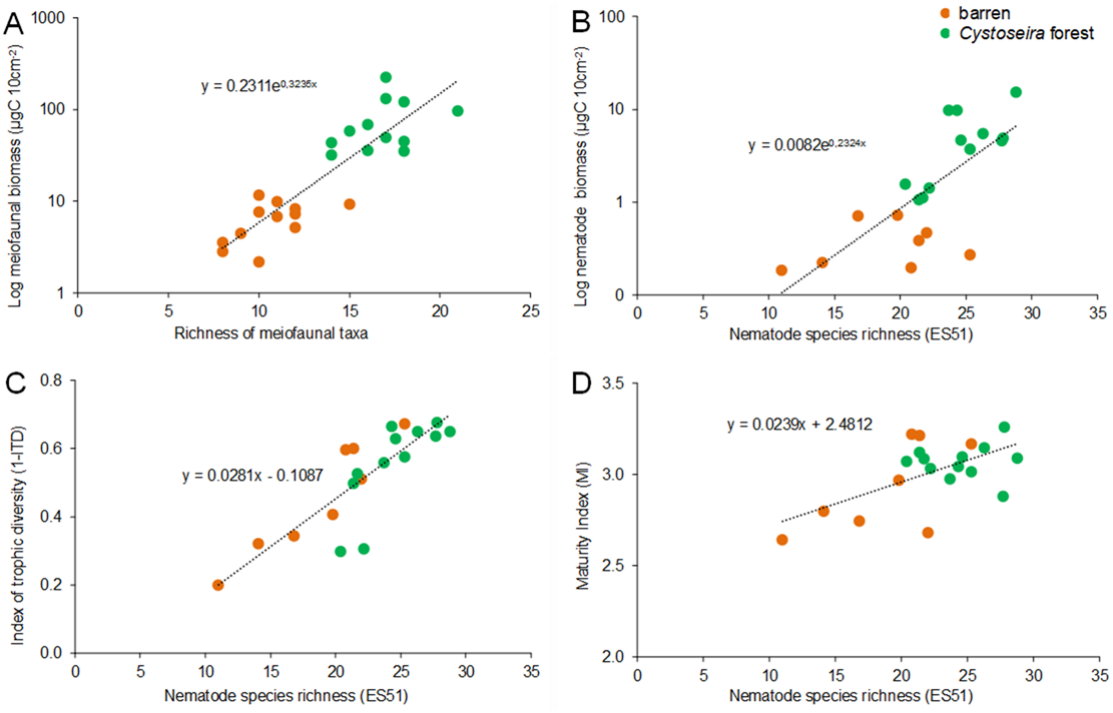
Relationships between biodiversity and ecosystem functioning and life-history attributes. Illustrated are the relationships between richness of meiofaunal higher taxa and biomass (**A**); nematode diversity as ES(51) and biomass (**B**); nematode diversity as ES(51) and Index of Trophic Diversity 1-ITD (**C**); nematode diversity as ES(51) and Maturity Index MI (**D**) in *Cystoseira* spp. forests and barren grounds. R^2^ values are reported in Table 3. P < 0.01 for all linear regressions.

**Figure 3:**
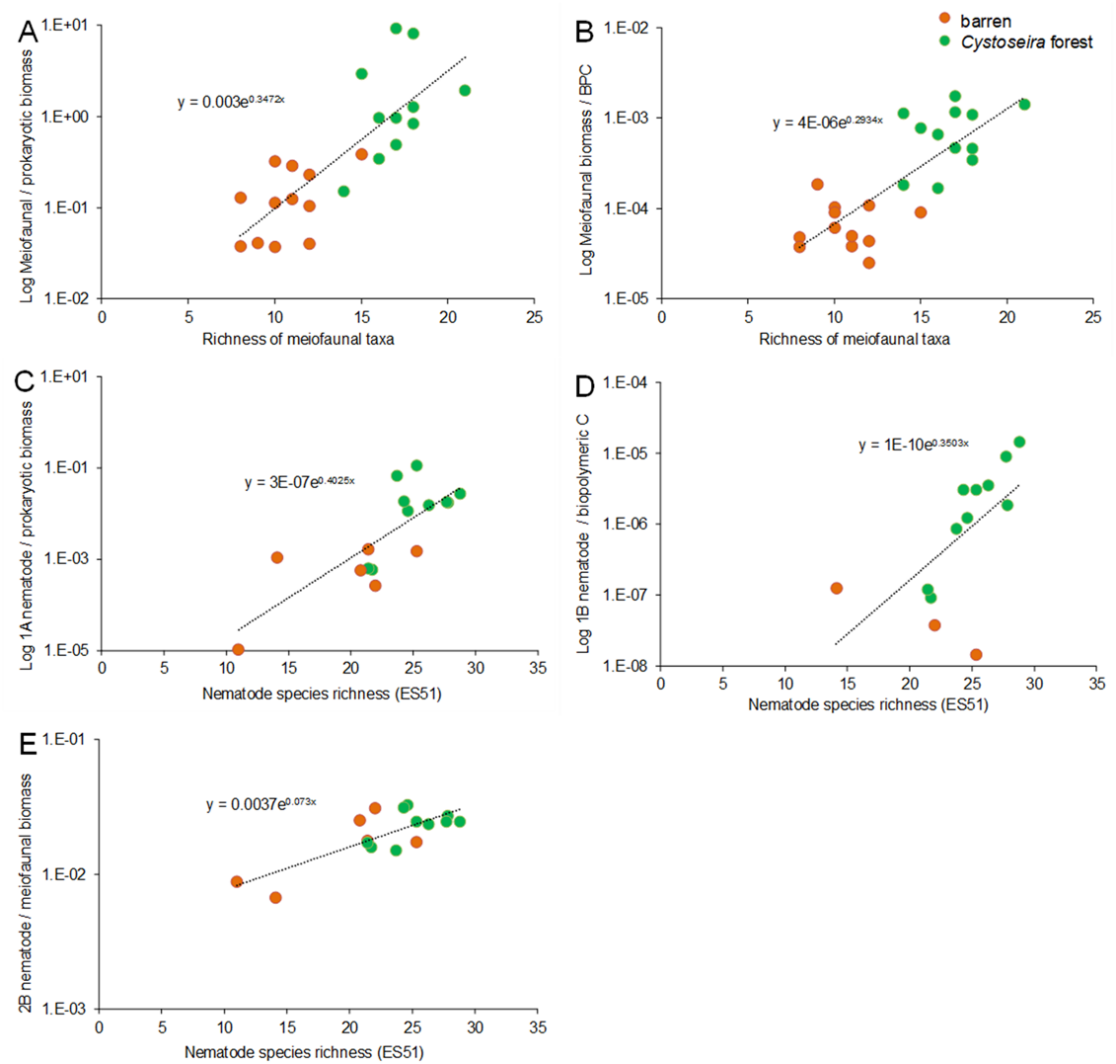
Relationships between biodiversity and ecosystem efficiency. Illustrated are the relationships between richness of meiofaunal higher taxa and meiofaunal/prokaryotic biomass ratio (**A**); richness of meiofaunal higher taxa and meiofaunal biomass/biopolymeric C ratio (**B**); nematode diversity as ES(51) and 1A-nematodes/prokaryotic biomass ratio (**C**); nematode diversity as ES(51) and 1B-nematodes biomass/biopolymeric C ratio (**D**) and nematode diversity as ES(51) and 2B-nematodes biomass/meiofaunal biomass ratio (**E**) in *Cystoseira* spp. forests and barren grounds. R^2^ values are reported in Table 3. P < 0.01 for all linear regressions.

**Figure 4:**
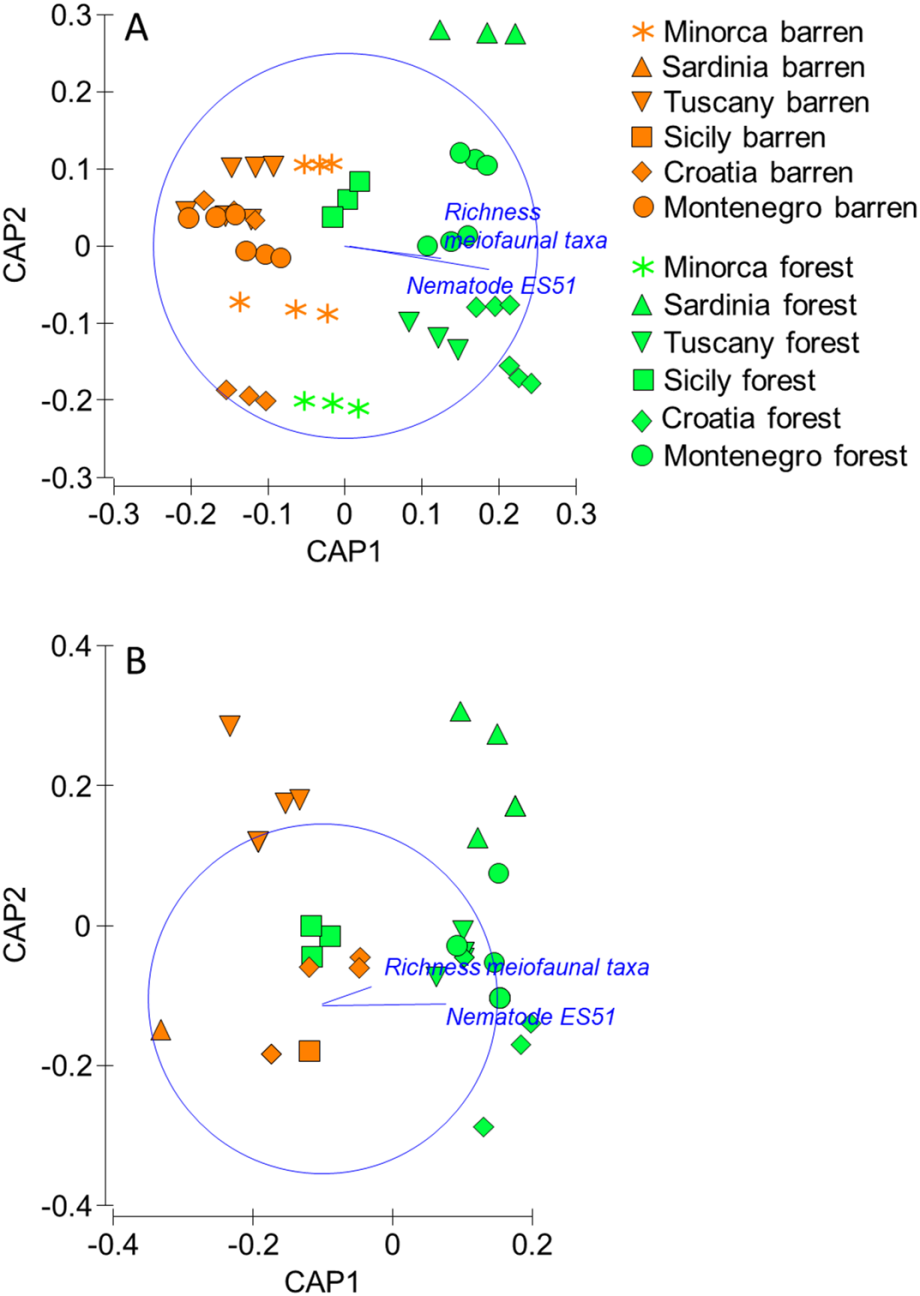
Output of CAP conducted on the ecosystem functioning (**A**) and efficiency (**B**). Vectors overlapped illustrate the variation of meiofaunal and nematode diversity as richness of higher taxa and nematode ES(51).

Regressions evaluating relationships between diversity (higher taxonomic or species level) and functional/trophic diversity, life strategies, ecosystem functioning and efficiency are reported in Table 3; Figs. 2A–D, 3A–E). The biplot after the CAP analyses revealed that differences between *Cystoseira* spp. forests and barren grounds in ecosystem functioning and efficiency are associated with both meiofaunal and nematode diversity, with the same pattern (Fig. 4A,B).

**Table 3:** Regression analyses of meiofaunal and nematode diversity against variables describing ecosystem functioning and efficiency. ***P < 0.001, **P < 0.01, *P < 0.05.

	Regression	R^2^	F	P
**Biodiversity vs ecosystem functioning**				
Richness of taxa vs Log meiofaunal biomass	y = 0.2311e^0.3235x^	0.770	73.6	***
ES(51) vs Log nematode biomass	y = 0.0082e^0.2324x^	0.541	10.8	**
ES(51) vs 1-ITD	y = 0.0281x − 0.1087	0.714	45.0	***
ES(51) vs MI	y = 0.0239x + 2.4812	0.363	10.3	**
**Biodiversity vs ecosystem efficiency**				
Richness of taxa vs meiofaunal/prokaryotic biomass	y = 0.003e^0.3472x^	0.642	6.3	*
Richness of taxa vs meiofaunal biomass/biopolymeric C	y = 4E−06e^0.2934x^	0.627	21.4	***
ES(51) vs Log 1A-nematode biomass/prokaryotic biomass	y = 3E−07e^0.4025x^	0.620	22.8	***
ES(51) vs 1B-nematode biomass/biopolymeric C	y = 1E−10e^0.3503x^	0.372	6.5	*
ES(51) vs 2B-nematode biomass/meiofaunal biomass	y = 0.0037e^0.073x^	0.608	12.4	**

## Discussion

Our results reveal that areas characterised by a loss of macroalgal forests can be characterised by a collapse of benthic biodiversity, either in terms of functional traits or trophic diversity (Fig. 1A). The biodiversity loss was also associated with a decrease in the availability of trophic resources. Such changes were associated, in some areas, with a significantly lower richness of nematode species^36^. The loss of biodiversity of macro- and megabenthic components as a result of the transition from macroalgal forests into barren grounds (dominated by sea urchins) was reported both in the Mediterranean and in other oceanic regions^53,54^.

Empirical and theoretical studies suggest that biodiversity regulates the ecosystem functions that are responsible for the production of natural goods and services^45,55–57^. Our results suggest that the loss of *Cystoseira* habitats and the transition of algal forests to barren grounds are also associated with the loss of specific ecosystem functioning (in terms of collapse of prokaryotic and meiofaunal biomass) and thus possibly with the goods and services that these habitats provide.

So far, investigations on the relationships between biodiversity and ecosystem functioning have been carried out using manipulative field experiments comprising assembled model (including non-natural) communities to assess the amount and direction of changes in diversity on several ecosystem processes. Approaches based on meta-analyses have also shown a positive effect of species diversity on ecosystem processes, consistently across different trophic levels and ecosystems^45,58,59^. In this study, the relationships between the richness of meiofaunal taxa and the nematodes species diversity *vs* proxies of ecosystem functioning (meiofaunal and nematode biomass) are positive and even exponential across all investigated areas (Fig. 2). These results are similar to those recently reported from tropical habitats, where fish diversity is positively exponentially related to ecosystem functioing^60^, and similar to previous studies conducted on meiofaunal assemblages where positive linear relationships were observed^48^.

While on one hand the effects of biodiversity loss on ecosystem functions are expected, the negative effect of habitat and biodiversity loss on ecosystem efficiency also provides new clues, which are independent from the absolute biomass values (Fig. 3). For example, our analyses suggest for the first time that the shift from algal forests to barren grounds and the consequent loss in meiofaunal biodiversity are associated with a lower efficiency in the exploitation of available organic C sources. In addition, the presence of exponential relationships produces an amplified negative effect of habitat and biodiversity losses on ecosystem functions.

Barrens showed also differences in terms of life strategies of the benthic assemblages (Fig. 1A), as were dominated by opportunistic species, instead of persistent species (K strategists), which dominated algal forest habitats. In all investigated areas, all of the variables associated with ecosystem functioning were significantly lower in barren grounds when compared with *Cystoseira* spp. forests. This pattern was found in different biogeographic regions of the Mediterranean for a wide range of environmental and biological features^36,61^.

The effects of the loss of macroalgal forests on the ecosystem functions were clear in terms of biomass and diversity of total meiofauna and nematodes, whereas those on prokaryotes biomass were less evident, probably as a result of the high variability at small spatial scales (i.e., within each forest and barren ground) of the prokaryotic components. Prokaryotes and meiofauna play key roles in biogeochemical cycles and in the energy transfer to higher trophic levels, as meiofauna is the preferred component of the diet of juveniles of macrofauna and bentho-nekton^62–66^. As a result, we hypothesise that the loss of meiofaunal biomass could have a cascading impact on different benthic components. Similar shifts in ecosystem functioning were observed in other vegetated habitats, such as tropical mangroves^67–69^, as well as in kelp forests of temperate and cold regions^70^.

Overall, these findings suggest that in Mediterranean macroalgal forests, the loss of the habitat forming *Cystoseria* spp. may cause a loss of meiofaunal species and ecosystem functions (here measured in terms of prokaryotic and meiofaunal biomass). The transition from the algal forests to barren grounds, including when barrens are small patches instead of vast “deserts” is of particular concern for the sustainable provisioning of ecosystem services in coastal areas. In the Mediterranean Sea, the decline in *Cystoseira* spp. forests is accelerated by multiple stressors (e.g., urbanization, eutrophication and increasing sediment loads), including climate change. Our findings demonstrate that the habitat loss might have multiple impacts on different levels of biological organization^54,71^.

Recent studies have revealed that coastal areas are natural-capital treasures and provide important benefits and ecosystem services. Recently, the natural capital associated with *Cystoseira* spp.-dominated forests has been assessed, starting from the identification of ecosystem functions (i.e., refuge, biogeochemical cycles maintenance, food provisioning, water oxygenation and protection from physical agents) and related services (i.e., water quality regulation and ecotourism) provided^13^. Due to the ecological and economic importance of *Cystoseira* spp. forests in the Mediterranean coastal environments, further studies are needed to investigate their natural recovery and resilience time. These systems have a relatively low cost, when compared to others as coral reefs^72^, but with high potential benefit return.

Moreover, the temporal dynamics of *Cystoseira* species are not yet well understood. Although some populations are characterized by long-lasting resilience^20^, the low dispersal ability of most *Cystoseira* species limits the natural recovery of most populations and of vegetated habitats^21^. Ongoing projects indicate that the restoration of *Cystoseira* spp. forests is possible using non-destructive methods and generating self-sustaining populations^33^. We recommend that policy makers plan active restoration actions (e.g., reforestation) to limit the loss of the important ecosystem services that these species provide^35,73^.

## Methods

### Study areas and data collection

**Figure 5:**
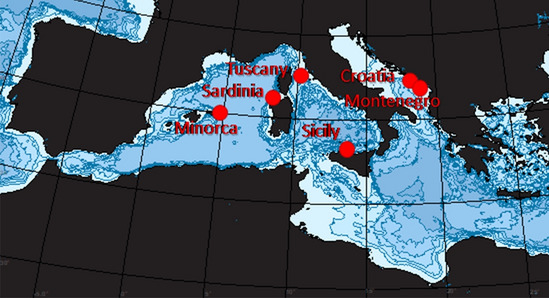
Location of the study areas in the Mediterranean Sea. The map was generated using Wikimedia Commons (CC BY-SA 4.0; https://commons.wikimedia.org/wiki/Category:Bathymetric_maps_of_the_Mediterranean_Sea#/media/File:Mediterranean_Sea_Bathymetry_map.svg, https://www.mathworks.com), and modified using Microsoft Power Point (Office 365 version).

Samples were collected in June–September 2014 from 6 areas spread over a longitudinal gradient in the western-central Mediterranean Sea (Fig. 5): Minorca (Spain), Sardinia, Tuscany, Sicily (Italy), Molunat (Croatia) and Tivat (Montenegro). The details of the sampling areas and activities were reported previously^36^. All areas were characterized by the presence of *Cystoseira* spp. forests and the presence of extended (Croatia and Montenegro) or patchy barren grounds (i.e., areas where was visually evident the loss of *Cystoseira* (i.e., Minorca, Sardinia, Tuscany and Sicily)^36,54^. In all of these areas, barren grounds were dominated by encrusting coralline algae and sea urchins, which typically characterise the rocks previously covered by algal forests^21,34,36,40,54,67^.

Samples were collected at 4–6 m depth by SCUBA divers, using a modified manual corer enabled to scrape the hard bottom surface^74^. Three replicate samples from 2 sites in each area were collected and immediately frozen at − 20 °C for prokaryotic biomass analyses. Analyses of C degradation rates were immediately carried out, assessing extracellular enzymatic activities^75^.

### Ecosystem functions

Three independent indicators of ecosystem functioning were considered: (i) prokaryotic biomass, (ii) total faunal biomass and (iii) organic matter decomposition. The use of biomass as a proxy of ecosystem functioning is widely consolidated in broad scientific studies, also beyond studies on marine ecosystems^47–52^.

Prokaryotic biomass for determined from cell counts carried out using epifluorescence microscopy after staining with SYBR green I and biovolume was estimated using a micrometer ocular assigning cells to different size classes, then converted into carbon content assuming 310 fg C mm^−3^^52^. For the determination of faunal biomass data were obtained from Bianchelli et al.^36^. For the measurement of organic matter decomposition, we determined extracellular enzymatic activities (as aminopeptidase) on surface sediments in triplicate by adding l-leucine-4-methylcoumarinyl-7-amide. Extracellular enzymatic activities (aminopeptidase and b-glucosidase) were determined by cleavage of artificial fluorogenic substrates (l-leucine-4-methylcoumarinyl-7-amide, Leu-MCA; 4-methylumbelliferone-b-d-glucopyranoside, Glu-MUF, respectively)^75^ at saturating concentrations. Incubations were performed in the dark at in situ temperature for 1 h. Then supernatants were analysed fluorometrically (at 380 nm excitation-440 nm emission for Leu-MCA and 365 nm excitation-455 nm emission for Glu-MUF). Fluorescence was converted into nmol of hydrolysed substrate using calibration curves obtained from standard solutions. The amount of hydrolyzed substrates was normalized to the incubation time and sediment dry weight (60 °C, 24 h), reported as nmol of substrate released g^−1^ h^−1^ and converted into equivalents of C mobilized (assuming 1 nmol of substrate hydrolyzed enzymatically corresponding to 72 ng of mobilized C), and their sum reported as C degradation rates^76^.

### Ecosystem efficiency

Ecosystem efficiency was calculated by determining the ratio of benthic faunal biomass to biopolymeric C content in the sediment. Biopolymeric C content estimated through the analysis of the biochemical composition of sediment organic matter and meiofaunal biomass were derived from Bianchelli et al.^36^.

### Data treatment and statistical analyses

Richness of meiofaunal higher taxa and nematodes ES51 were used as proxy of biodiversity. The 1-ITD and MI indexes were used as proxy of trophic/functional diversity and life strategies, respectively. BPC concentration in sediments was used as proxy of potential trophic resources available for prokaryotes and meiofauna, comprising nematodes^36,77^. Degraded C per prokaryotic cell, prokaryotic, meiofaunal and nematodes biomass were used as proxies of ecosystem functioning. Meiofaunal biomass to BPC and to prokaryotic biomass ratios, as well as 1A-nematodes to prokaryotic biomass, 1B-nematodes biomass to BPC and 2B-nematodes to meiofaunal biomass ratios were used as proxies of ecosystem efficiency. All data dealing with organic loads, meiofaunal and nematode diversity were extracted from a previous study^36^.

To assess differences between forests vs barren grounds for all the considered variables, univariate distance-based permutational analyses of variance (PERMANOVA^78^), after data log-transformation, was applied. All statistical analyses were carried out using the same experimental design, considering 2 factors as main sources of variance: State (fixed, 2 levels: forests and barren) and Site (random and nested in State, 2 levels: 1 and 2), separately for each investigated area, after ascertaining significant differences in dispersion among groups (PERMDISP; Supplementary Table S2). We used the PERMANOVA tests based on matrices of Euclidean distance for all the investigated variables. Univariate PERMANOVAs were carried out using the routines included in the software PRIMER 6+^79^.

Relationships between biodiversity and ecosystem functioning/efficiency variables were also investigated using regressions analysis, in order to evaluate the direction and scale of variations^50^.

To visualize the differences between forests and barrens for the investigated variables we first estimated the effect sizes with log–response ratios^80,81^: R_i_ = ln (X_Bi_/X_Mi_), where R_i_ is the log–response ratio for the response category (i.e., barren conditions) of the area i, and X_Bi_ and X_Mi_ are the mean values of the metric for area i in barrens (B) and meadows (M), respectively.

To visualize differences between states and areas in the overall ecosystem functioning and efficiency and to overlap the trends of diversity at higher taxa and species levels, bi-plots after a Canonical Analysis of Principal Coordinates (CAP) were also prepared^82^.

## Supplementary information

Supplementary Tables.
