# Supplementary material for: Impairment of microbial and meiofaunal ecosystem functions linked to algal forest loss
Source: Sci Rep. 2020 Nov 17;10:19970. doi: 10.1038/s41598-020-76817-5 (PMC7673138; doi:10.1038/s41598-020-76817-5)
Supplement: Supplementary file 1 — Supplementary Tables. [file 41598_2020_76817_MOESM1_ESM.pdf]

Impairment of microbial and meiofaunal ecosystem functions linked to algal forest loss

Silvia Bianchelli [1,*] **and Roberto Danovaro** [1,2]

[1] Dipartimento di Scienze della Vita e dell'Ambiente, Università Politecnica delle Marche, Via Brecce Bianche, 60131 Ancona, Italy.

[2] Stazione Zoologica Anton Dohrn di Napoli, Villa Comunale, 80121 Napoli, Italy.

*Corresponding author: silvia.bianchelli@univpm.it

Supplementary Table S1. Results of PERMANOVA testing variations between states (forests vs barren grounds) in the C degraded per prokaryotic cell, prokaryotic and nematodes biomass (A) and in meiofaunal to prokaryotic biomass and biopolymeric C ratios, as well as 1A-nematodes to prokaryotic biomass, 1B-nematodes to biopolymeric C and 2B-nematodes to meiofaunal biomass ratios (B). dF=degree of freedom; MS=mean square; F=F statistic; ***=P < 0.001; ** =P < 0.01; * =P < 0.05; na=not available; ns=not significant.

			Source	df	MS	F	P	% explained variance
**A)**	**C degraded per prokaryotic cell**	Minorca	State	1	5.65	1.88	ns	37.7
			Site(State)	1	3.00	20.82	**	54.1
			Residual	6	0.14			
		Sardinia	na					
		Tuscany	State	1	0.37	4.10	ns	43.0
			Site(State)	2	0.09	1.90	ns	13.1
			Residual	8	0.05			
		Sicily	na					
		Croatia	State	1	1.76	0.58	ns	15.1
			Site(State)	2	3.02	11.72	**	66.4
			Residual	8	0.26			
		Montenegro	State	1	0.17	0.03	ns	30.9
			Site(State)	2	5.09	23.99	**	61.2
			Residual	8	0.21			
	**Total prokaryotic biomass**	Minorca	State	1	0.55	1.58	ns	30.4
			Site(State)	1	0.35	326.22	***	69.0
			Residual	6	0.00			
		Sardinia	State	1	3.30	8.94	ns	79.8
			Site(State)	2	0.37	414.40	***	20.1
			Residual	8	0.00			
		Tuscany	State	1	0.43	4.45	ns	61.9
			Site(State)	2	0.10	33.27	***	34.8
			Residual	8	0.00			
		Sicily	State	1	0.76	6.32	ns	72.6
			Site(State)	2	0.12	531.61	***	27.2
			Residual	8	0.00			
		Croatia	State	1	0.05	0.11	ns	30.8
			Site(State)	2	0.48	554.73	***	68.8
			Residual	8	0.00			
		Montenegro	State	1	0.06	0.26	ns	27.0
			Site(State)	2	0.23	344.61	***	72.3
			Residual	8	0.00			
	**Nematodes biomass**	Minorca	State	1	0.34	115.57	*	63.0
			Site(State)	2	0.00	0.12	ns	8.4
			Residual	8	0.03			
		Sardinia	State	1	7.74	45.99	*	94.0
			Site(State)	2	0.17	4.66	*	3.3
			Residual	8	0.04			
		Tuscany	State	1	5.41	283.32	**	97.6
			Site(State)	2	0.02	0.89	ns	0.1
			Residual	8	0.02			
		Sicily	State	1	5.77	8989.90	***	93.7
			Site(State)	2	0.00	0.01	ns	1.6
			Residual	8	0.05			
		Croatia	State	1	7.53	67.91	*	96.4
			Site(State)	2	0.11	7.91	*	2.5
			Residual	8	0.01			
		Montenegro	State	1	4.62	66.58	*	95.9
			Site(State)	2	0.07	5.05	*	2.3
			Residual	8	0.01			

B)	**Meiofaunal biomass/prokaryotes biomass ratio**	Minorca	State	1	0.13	0.16	ns	37.1
			Site(State)	1	1.07	26.32	**	56.2
			Residual	5	0.04			
		Sardinia	State	1	17.35	102.65	*	85.3
			Site(State)	2	0.17	0.41	ns	2.4
			Residual	8	0.41			
		Tuscany	State	1	1.39	10.35	***	67.4
			Site(State)	2	0.13	1.58	ns	5.3
			Residual	8	0.08			
		Sicily	State	1	2.29	26.32	*	65.1
			Site(State)	2	0.09	0.51	ns	4.9
			Residual	8	0.17			
		Croatia	State	1	3.72	7.12	***	73.3
			Site(State)	2	0.52	17.94	**	22.6
			Residual	8	0.03			
		Montenegro	State	1	4.27	53.57	*	93.9
			Site(State)	2	0.08	2.85	ns	2.3
			Residual	8	0.03			
	**Meiofaunal biomass/biopolymeric C ratio**	Minorca	State	1	0.95	3.68	ns	49.3
			Site(State)	2	0.26	5.29	*	29.8
			Residual	8	0.05			
		Sardinia	State	1	4.34	22.70	*	88.8
			Site(State)	2	0.19	5.40	*	6.7
			Residual	8	0.04			
		Tuscany	State	1	2.67	34.37	*	90.7
			Site(State)	2	0.08	2.79	ns	3.5
			Residual	8	0.03			
		Sicily	State	1	1.12	17.01	*	81.0
			Site(State)	2	0.07	2.27	ns	5.7
			Residual	8	0.03			
		Croatia	State	1	3.59	27.85	*	64.9
			Site(State)	2	0.13	0.48	ns	5.1
			Residual	8	0.27			
		Montenegro	State	1	5.01	233.43	**	97.5
			Site(State)	2	0.02	0.99	ns	0.0
			Residual	8	0.02			
	**1A-nematodes biomass/prokaryotic biomass ratio**	Minorca	na					
		Sardinia	State	1	32.42	2053.30	*	99.5
			Site(State)	1	0.02	0.46	ns	0.1
			Residual	6	0.03			
		Tuscany	State	1	4.67	67.73	**	93.4
			Site(State)	2	0.07	1.48	ns	0.9
			Residual	8	0.05			
		Sicily	State	1	0.80	105.18	**	84.1
			Site(State)	1	0.01	0.25	ns	3.2
			Residual	6	0.03			
		Croatia	State	1	7.40	33.01	*	89.3
			Site(State)	2	0.22	2.19	ns	3.0
			Residual	8	0.10			
		Montenegro	State	1	4.50	60.30	*	94.6
			Site(State)	2	0.07	2.86	ns	2.1
			Residual	8	0.03			
	**1B-nematodes biomass/biopolymeric C ratio**	Minorca	na					
		Sardinia	State	1	10.14	80.22	**	97.0
			Site(State)	2	0.14	4.76	ns	1.9
			Residual	6	0.03			
		Tuscany	State	1	17.29	220.59	**	95.5
			Site(State)	2	0.08	0.65	ns	0.5
			Residual	8	0.12			
		Sicily	State	1	0.13	4.04	***	52.2
			Site(State)	2	0.03	2.75	ns	20.9
			Residual	6	0.01			

	Croatia	State	1	11.91	27.98	*	91.5
		Site(State)	2	0.43	7.96	**	5.9
		Residual	8	0.05			
	Montenegro	State	na				na
		Site(State)	1	0.36	6.10	ns	63.0
		Residual	4	0.06			
**2B-nematodes biomass /meiofaunal biomass ratio**	Minorca	na					
	Sardinia	State	1	1.07	55.50	*	69.1
		Site(State)	2	0.02	0.30	ns	5.8
		Residual	8	0.06			
	Tuscany	State	1	0.23	12.20	*	21.9
		Site(State)	2	0.02	0.09	ns	10.3
		Residual	8	0.20			
	Sicily	State	1	0.58	39.88	*	54.6
		Site(State)	2	0.01	0.23	ns	9.3
		Residual	8	0.06			
	Croatia	State	1	0.18	1.08	ns	2.6
		Site(State)	2	0.17	4.46	ns	52.2
		Residual	8	0.04			
	Montenegro	State	1	0.00	0.00	ns	3.4
		Site(State)	2	0.07	0.26	ns	19.0
		Residual	8	0.27			

Supplementary Table S2. Results of PERDISP testing for homogeneity of multivariate dispersions among areas, between states (forests vs barren grounds) and between states separately for each area in the C degraded per prokaryotic cell, prokaryotic and nematodes biomass and in meiofaunal to prokaryotic biomass and biopolymeric C ratios, as well as 1A-nematodes to prokaryotic biomass, 1B-nematodes to biopolymeric C and 2B-nematodes to meiofaunal biomass ratios. ***=P < 0.001; ** =P < 0.01; * =P < 0.05; na=not available; ns=not significant.

		C degraded per prokaryotic cell		Total prokaryotic biomass		Nematodes biomass		Meiofaunal biomass/ prokaryotes biomass ratio		Meiofaunal biomass/ biopolymeric C ratio		1A-nematodes biomass/ prokaryotic biomass ratio		1B-nematodes biomass/ biopolymeric C ratio		2B-nematodes biomass / meiofaunal biomass ratio	
		f	P	f	P	f	P	f	P	f	P	f	P	f	P	f	P
Among areas		2.21	ns	5.18	***	18.07	***	7.36	***	3.02	***	2.65	*	10.62	***	0.45	ns
Between states		0.69	ns	0.91	ns	0.03	ns	0.01	ns	2.59	ns	0.01	ns	0.004	ns	5.16	*
In each area between states	Minorca	4.50	*	94.20	*	0.55	ns	2.24	ns	1.45	ns	na	na	na	na	na	na
	Sardinia	na	na	113.28	**	0.57	ns	0.03	ns	2.35	ns	0.46	ns	0.57	ns	0.90	ns
	Tuscany	0.003	ns	6.26	*	0.42	ns	0.01	ns	0.74	ns	0.43	ns	0.16	ns	0.01	ns
	Sicily	na	na	493.70	**	4.63	*	0.36	ns	0.03	ns	1.39	ns	0.00	ns	1.77	ns
	Croatia	6.65	*	409.96	**	1.11	ns	10.34	**	0.75	ns	0.19	ns	0.22	ns	1.63	ns
	Montenegro	16.91	**	317.36	**	2.21	ns	0.77	ns	0.51	ns	0.89	ns	na	na	0.09	ns
